# Human Blood-Derived lncRNAs in Autism Spectrum Disorder

**DOI:** 10.3390/biom15070937

**Published:** 2025-06-27

**Authors:** Carmela Serpe, Paola De Sanctis, Marina Marini, Silvia Canaider, Provvidenza Maria Abruzzo, Cinzia Zucchini

**Affiliations:** Department of Medical and Surgical Sciences, University of Bologna, Via Massarenti 9, 40138 Bologna, Italy; carmela.serpe@unibo.it (C.S.); paola.desanctis@unibo.it (P.D.S.); silvia.canaider@unibo.it (S.C.); cinzia.zucchini@unibo.it (C.Z.)

**Keywords:** long non-coding RNA, lncRNA, autism spectrum disorder, ASD, biomarkers, non-coding RNA, peripheral blood

## Abstract

Autism spectrum disorder (ASD) is a complex and heterogeneous neurodevelopmental disorder with a significant impact on public health. ASD diagnosis is based on clinical observation and typically occurs around three years of age. The identification of reliable ASD markers could facilitate early diagnosis and help pinpoint therapeutic targets for effective interventions. Long non-coding RNAs (lncRNAs), particularly those derived from blood, have been recently proposed as potential biomarkers in many pathological conditions, including neurological diseases. This manuscript summarizes original studies examining human dysregulated blood-derived lncRNAs as potential ASD biomarkers. LncRNAs are described by grouping them according to the selection strategy used by the authors: (i) lncRNAs involved in biological processes impaired in ASD or in pathological conditions sharing the disrupted signaling pathways of ASD; and (ii) lncRNAs identified through high-throughput analysis. The study highlights key priorities for future research: assessing the ability of lncRNAs to distinguish ASD from other neurological disorders, extending analyses to larger and younger cohorts to validate candidate biomarkers in early life, and integrating multiple data sources to establish validated biomarker networks for clinical application. This review indicates that research on blood-derived lncRNAs in ASD is still in its early stages.

## 1. Introduction

Autism spectrum disorder (ASD) is a complex and heterogeneous neurodevelopmental disorder with a significant impact on public health [1]. It is characterized by deficits in social interaction and communication, along with repetitive and restrictive behaviors [2]. In addition to these core symptoms, ASD is frequently associated with various comorbidities, including intellectual disability, depressive disorders, sleep disturbances, and gastrointestinal and immunological abnormalities [3]. Moreover, ASD subjects often experience additional health concerns, such as epilepsy, electroencephalogram abnormalities, dysmorphic features, and atypical magnetic resonance imaging findings [4,5,6]. The prevalence of ASD has markedly increased worldwide in the last few decades. According to the CDC’s Autism and the Developmental Disabilities Monitoring (ADDM) Network, 1 in 36 children aged 8 years in the United States is currently diagnosed with ASD, with a male-to-female ratio of approximately 4:1 [7]. The increase in ASD prevalence over time results from a complex interplay of factors, including improvements in diagnostic criteria and greater awareness of the disorder [8]. ASD is diagnosed by expert clinicians around the age of 3 years [9], using standardized diagnostic tools such as the Autism Diagnostic Observation Schedule (ADOS), the Childhood Autism Rating Scale (CARS), and the Autism Diagnostic Interview—Revised (ADI-R) to assess children’s behavior and symptoms [10,11]. However, due to the highly heterogeneous nature of ASD, this approach often delays ASD diagnosis [12]. Although numerous biological, physiological, and medical alterations have been identified in ASD, none of these are used as a diagnostic marker to aid early diagnosis, treatment, and management [13,14]. The lack of reliable biomarkers is primarily due to the limited understanding of ASD etiology. To date, ASD is considered a multifactorial disorder caused by a complex interplay of genetic and environmental factors [15]. Several studies, including twin studies, have shown that genetic factors greatly contribute to ASD susceptibility [16]. Common and rare genetic alterations, including de novo and rare copy number variations (CNVs) and single nucleotide variations (SNVs), have been found in several genes associated with chromatin remodeling, neural development, synaptic function, and neuronal communication, significantly contributing to ASD risk [17,18]. However, DNA alterations account for only 35–40% of ASD cases, suggesting that other factors are required for ASD onset. Environmental factors, including prenatal and perinatal causes, and microbial–gut–brain axis alterations are implicated in ASD etiology [19]. These factors may directly affect the transcription of specific susceptibility genes through epigenetic modifications, such as DNA methylation, chromatin remodeling, and alterations in the expression of non-coding RNA molecules, like microRNAs (miRNAs) and long non-coding RNAs (lncRNAs).

The recent advent of next-generation sequencing (NGS) has revealed that 85% of the human genome is actively transcribed, with only 2–3% of these transcripts coding for proteins [20,21]. The huge amount of genome containing information for non-coding RNA (ncRNA) transcripts indicates that these molecules might have wide and diversified roles in biological processes. Based on their size, ncRNAs are classified into two main groups: short ncRNAs, which are less than 200 nucleotides long, and long non-coding RNAs (lncRNAs), which are longer than 200 nucleotides. Short ncRNAs include miRNAs, piwi-interacting RNAs (piRNAs), circular RNAs (circRNAs), and small nucleolar RNAs (snoRNAs). LncRNAs are the most abundant class of ncRNAs in the human genome [20]. They are mainly transcribed by RNA polymerase II (PolII) and share characteristics with coding RNA genes, such as 7-methylguanosine cap, polyA tail, and alternative splicing [21,22]. Most lncRNAs overlap with protein-coding loci and can be divided into sense, antisense, and intronic lncRNAs. Additionally, some lncRNAs are located in the intergenic regions of the genome (lincRNAs) [21,22]. LncRNAs exert their functions in both the nucleus and cytoplasm, where they regulate gene expression at multiple levels by interacting with RNA, DNA, and proteins [23,24]. For instance, lncRNAs function as epigenetic regulators by acting as scaffolds for histone-modifying complexes and DNA methyltransferases or demethylases, thus modulating chromatin architecture [22,25,26]. LncRNAs also regulate transcriptional and post-transcriptional processes by interacting directly with transcription factors and proteins involved in mRNA maturation and alternative splicing [26,27,28,29]. In addition, lncRNAs can interact with miRNAs by acting as miRNA sponges or competing endogenous RNAs (ceRNAs) [26,30,31]. Finally, lncRNAs are also implicated in translational and post-translational gene expression control [26,29,32,33]. This scenario highlights the complex role of these molecules in the regulation of several biological pathways. LncRNAs are implicated in a wide variety of physiological processes, including proliferation and differentiation, metabolism, signaling pathways, and apoptosis [34]. The abnormal expression of lncRNAs, observed in many diseases [35,36,37,38,39], has attracted the attention of researchers seeking novel biomarkers. LncRNAs, and in particular, blood-derived lncRNAs, have been recently proposed as potential biomarkers in many pathological conditions, including cancer, neurological diseases, and ASD [40,41,42,43,44,45]. Their high specificity, stability, and relative abundance as well as cost-effectiveness and accessibility make them attractive diagnostic and prognostic tools for clinical applications [40].

**Figure 1:**
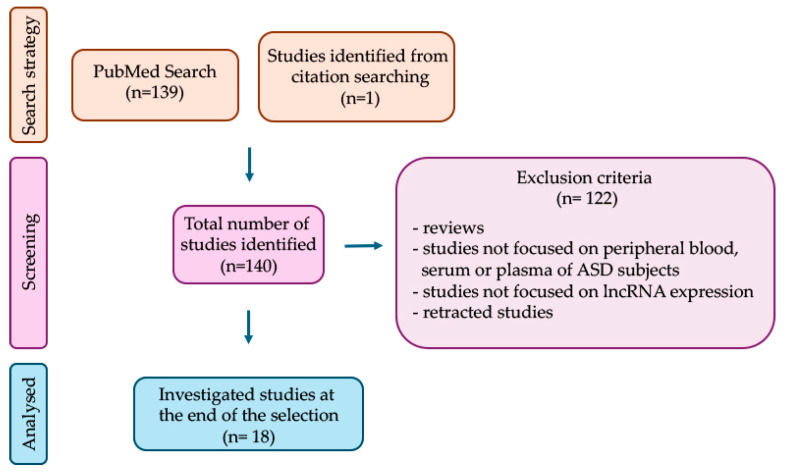
Study selection process. The flow diagram shows the number of investigated studies identified (n), according to the declared criteria.

This is a narrative review summarizing original studies that have examined dysregulated blood-derived lncRNAs in ASD as possible biomarkers. For this purpose, a search was performed on PubMed up to 1 September 2024 using the search terms (lncRNA), (long non-coding RNA) in combination with the terms (autism), (autism spectrum disorder). Among the retrieved original articles, only those that measured lncRNA expression levels in peripheral blood, serum, or plasma of ASD subjects, compared to controls, were considered eligible. This screening, carried out by two independent reviewers through reading the title, abstract, and materials and methods sections, led to the selection of 18 articles. Additional information was retrieved from the analysis of the reference list of the selected articles (Figure 1). Finally, the relevance of the selected studies was assessed by reading the full text. The following sections summarize dysregulated lncRNAs in ASD identified in one or more studies, along with details on their diagnostic power.

## 2. Blood-Derived lncRNAs Dysregulated in Autism Spectrum Disorder (ASD)

The literature review revealed that authors employed two main approaches to identify dysregulated lncRNAs. A common strategy is based on the selective choice of lncRNAs according to their involvement in biological processes impaired in ASD or in pathological conditions that share the same disrupted signaling pathways with ASD. As an alternative approach, other authors have employed high-throughput analysis, which offers the advantage of detecting many dysregulated lncRNAs simultaneously, irrespective of their putative role in the disease. Therefore, in the following sections, blood-derived lncRNAs are described by grouping them according to the above-mentioned selection strategies.

### 2.1. LncRNAs Selected According to ASD-Related Pathways

Alterations in neurological processes during neurogenesis, including synaptic function, neuronal activity, neuronal cell adhesion, neurite growth, and synaptogenesis, are well-established contributors to ASD pathogenesis. Genes involved in these processes are among the risk loci associated with ASD [46]. Mounting evidence shows that lncRNAs are mainly expressed in the nervous system; therefore, it is not surprising that many lncRNAs have received attention as candidate biomarkers [47,48]. It is known that the Disrupted in schizophrenia 2 (DISC2) lncRNA, located on human chromosome 1q42 and transcribed antisense to exon 9 of the gene *DISC1* [49,50], plays a role in neuronal differentiation [51]. A deletion of chromosome 1q42, which includes *DISC2*, has been described in a 3-year-old male child affected by ASD [52]. However, the involvement of this lncRNA in ASD pathogenesis remains unclear, since contradictory results are reported. Tamizkar and colleagues [53] found an increased expression of DISC2 in the peripheral blood of ASD children compared to controls, while, more recently, Raahmani and colleagues [54] observed no significant difference in the expression levels of DISC2, suggesting that further investigation is needed. In addition to DISC2, Tamizkar and colleagues [53] observed alterations in the expression of three other lncRNAs: PRKAR2A antisense RNA 1 (PRKAR2A-AS1), LINC03091 long intergenic non-protein coding RNA 3091 (LINC03091 or LOC101928237), and LRRC2 antisense RNA 1 (LRRC2-AS1). In particular, the authors found an increase in the expression of PRKAR2A-AS1 and LOC101928237 and a decrease in LRRC2-AS1 levels in ASD children. Although the specific function of these lncRNAs in ASD is still unknown, the authors suggest that they may contribute to ASD pathogenesis and could be used as potential markers. Indeed, LOC101928237 and LRRC2-AS1 show a strong ability in distinguishing ASD from control children (AUC: 0.90 and 0.929, respectively) (Table 1). The authors also highlighted the need to validate these results in a larger cohort of subjects and to investigate these lncRNA signatures during the early stages of ASD pathogenesis [53].

The Small nucleolar RNA host gene 6 (SNHG6) lncRNA is related to the vitamin D receptor (VDR) pathway [55], which affects neurodevelopment, axon connectivity, and dopamine formation [56]. Ghafouri-Fard and collaborators [57] found a downregulation in SNHG6 expression in the blood of ASD subjects compared to the control group. A possible molecular mechanism comes from a previous study showing that SNHG6 can suppress miR-181c [58], which was found overexpressed in the amygdala of an ASD rat model [59]. Therefore, the authors highlighted the role of the SNHG6/miR-181c axis as a potential functional mechanism in the development of ASD. Moreover, based on Receiver Operating Characteristic Curve (ROC) analysis, which revealed high performance in discriminating ASD cases from controls, the authors proposed SNHG6 as a potential biomarker (AUC: 0.94) (Table 1).

Recently, Sane and colleagues [60] evaluated in blood samples of ASD children and healthy subjects the expression levels of five lncRNAs involved in the modulation of neurological response and in the differentiation of neuronal cells: the Long intergenic non-protein coding RNA, regulator of reprogramming (LincRNA-ROR), Long intergenic non-protein coding, p53-induced transcript (LINC-PINT), Long intergenic non-coding RNA p21 (LincRNA-p21), Prostate cancer-associated transcript 29 (PCAT-29), and Prostate cancer-associated transcript-1 (PCAT-1). The study revealed that all the examined lncRNAs were significantly downregulated in individuals with ASD compared to the healthy controls. ROC analysis indicated that LincRNA-ROR and PCAT-1 had better diagnostic power than PCAT-29, LINC-PINT, and LincRNA-p21 in distinguishing ASD from healthy subjects (Table 1) [60].

**Figure 2:**
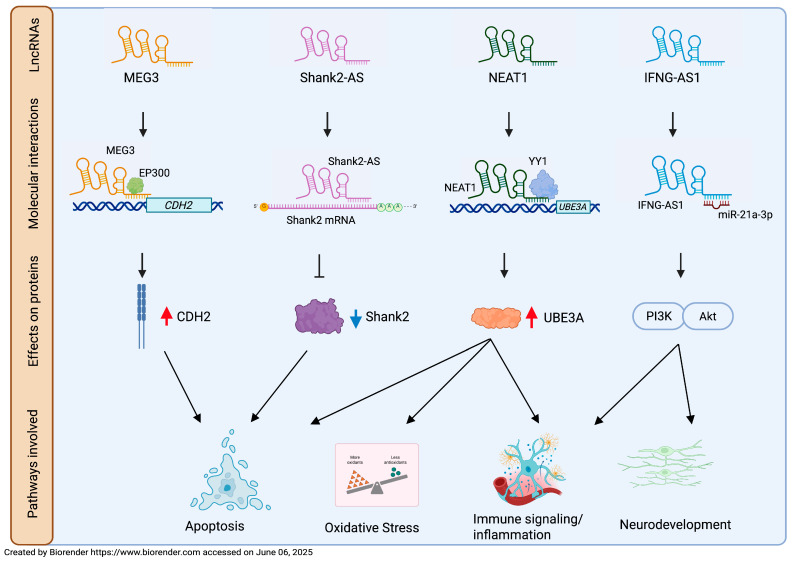
LncRNA-affected molecular mechanism in ASD pathogenesis. A schematic representation of molecular mechanisms observed for MEG3, NEAT1, Shank2-AS, and IFNG-AS1 in ASD cellular and animal models. CDH2, cadherin2; EP300, E1A binding protein p300 transcription factor; YY1, Yin-Yang 1 transcription factor; UBE3A, ubiquitin protein ligase E3A; PI3K/AKT, phosphoinositide 3-kinase/AKT serine/threonine kinase; ⊥ means inhibition; ↓ means promotion; red arrow means increased expression; blue arrow means decreased expression.

The Maternally expressed gene 3 (MEG3) lncRNA, encoded by an imprinted gene located on human chromosome 14q32.3, regulates AMPA receptor surface expression and is involved in neuronal synaptic plasticity [61]. Taheri and colleagues [62] found a significant upregulation of MEG3 lncRNA in the blood of ASD children compared to controls and suggested MEG3 as a putative biomarker, according to ROC analysis (AUC 0.792) (Table 1). Recent findings indicated that MEG3 is also involved in the apoptotic pathway in the hippocampal tissues of valproic acid (VPA)-treated rats; in this model, MEG3 is overexpressed and promotes cadherin2 (CDH2) expression via E1A binding protein p300 transcription factor (EP300), repressing neuronal viability [63] (Figure 2).

Apoptosis is one of the most explored biological pathways to discover new candidate markers for ASD. Apoptosis plays a crucial role in eliminating excess neurons and refining neural circuits during brain development. Its dysregulation affects neuronal density, number, organization, and composition, leading to impaired connectivity and synaptic dysfunctions [64]. Aberrant regulation of apoptotic pathways has been observed in both postmortem brain tissue and peripheral blood of subjects with ASD [65,66]. The antisense lncRNA of SH3 and multiple ankyrin repeat domains 2 (Shank2-AS) is reversely transcribed from the 10th intron of the *SHANK2* gene, a locus associated with ASD [67,68]. Its overexpression decreases neurite length and numbers, inhibits the proliferation of neuronal cells, and promotes their apoptosis by downregulating Shank2 protein expression by forming a double-stranded RNA [69] (Figure 2). Luo and colleagues [69] found an upregulation of Shank2-AS in lymphocytes from ASD peripheral blood. This result is consistent with the Shank2-AS overexpression previously observed by Wang and colleagues in a high-throughput study conducted on ASD leukocytes, which is described below [70]. These findings suggest that targeting lncRNAs located at ASD-causing loci may be a promising approach in the search for new putative biomarkers of the disease. Unfortunately, although the concordance of Shank2-AS dysregulation in two independent cohorts of subjects supports its potential role as a candidate biomarker, the authors did not provide data regarding its diagnostic utility.

The Nuclear paraspeckle assembly transcript 1 (NEAT1) lncRNA, located on chromosome 11q13.7, was originally investigated by Sayad and colleagues [71] for its regulatory role in apoptosis in colon cancer [72]. This apoptotic function was subsequently confirmed in many neurological diseases, including ASD [73,74]. In summary, NEAT1 promotes apoptosis, oxidative stress (OS) and inflammation in VPA-induced ASD rats by recruiting Yin-Yang 1 (YY1) transcription factor to regulate ubiquitin protein ligase E3A (UBE3A) expression [74] (Figure 2). A significant increase in NEAT1 levels was observed in the peripheral blood of ASD patients compared to controls. The authors proposed that NEAT1 might contribute to ASD pathogenesis via the miR-497/brain-derived neurotrophic factor (BDNF) pathway, similarly to what has been observed in diabetic retinopathy; this pathological condition is characterized by NEAT1 downregulation, which, in turn, decreases BDNF expression through the upregulation of miR-497 and leads to the death of Muller cells [75]. Conversely, increased NEAT1 is in line with the elevated peripheral BDNF levels in ASD subjects, thus supporting the involvement of miR-497/BDNF in ASD [71,76]. Although the precise mechanism is still unclear and warrants further investigation, NEAT1 has been proposed by Sayad and collaborators [71] as a potential diagnostic biomarker for ASD based on its AUC value (AUC: 0.759) (Table 1). The same research group also found a significant overexpression of Taurine upregulated gene 1 (TUG1) lncRNA in the peripheral blood of children with ASD compared to controls. Moreover, according to ROC analysis, TUG1 could acceptably distinguish ASD from healthy subjects (AUC: 0.733) (Table 1) [71]. Several authors have highlighted the apoptotic role of TUG1 exerted by its sponge activity in many neuronal dysfunctions [77,78,79,80]. Although Sayad and collaborators [71] did not explore the mechanism by which TUG1 would be implicated in the pathogenesis of ASD, they suggested that TUG1 might act as a sponge for miR9. This apparently contrasts with the finding that, in a knockdown cell model, the lncRNA TUG1 acts as a positive regulator of TBC/LysM-associated domain containing 1 (TLDC1) [81], a member of the TLDc family proteins that exerts a protective function against OS. Consistent with the findings reported by Sayad and colleagues [71], Zucchini and collaborators [82] found that TUG1 expression level was increased in the Peripheral Blood Mononuclear Cells (PBMCs) of ASD children, thus reinforcing the implication of TUG1 in this disorder (Table 1). Moreover, the concurrent increase in TLDC1 mRNA level suggests a role of TUG1 in protection against OS, a key factor in the ASD pathophysiology [83,84,85].

A neuroprotective function has also been proposed for the Plasmacytoma variant translocation 1 (PVT1) lncRNA [86]. Its expression is altered in some neurological disorders, such as epilepsy [87] and schizophrenia [88,89]. Jiang and colleagues [90] found that PVT1 was downregulated in the serum of children with ASD compared to healthy controls. ROC analysis indicated that PVT1 may be a useful biomarker for early ASD screening, either alone (AUC: 0.848, sensitivity: 85% and specificity: 79.3%) (Table 1) or in association with miR-21-5p, one of its putative targets [91]. Interestingly, combining both PVT1 with miR-21-5p yielded higher diagnostic performance than either marker alone (AUC: 0.954, sensitivity: 83.3%, and specificity: 96.6%) [90], reinforcing the idea that using functionally related markers can improve diagnostic accuracy.

Mitochondrial alterations not only increase the production of Reactive Oxygen Species (ROS) and Reactive Nitrogen Species (RNS) but also impair energy metabolism and calcium homeostasis regulation [92,93]. Several lines of evidence support the crucial role of mitochondrial dysfunction and calcium imbalance in ASD [94,95,96,97]. Pourtavakoli and collaborators [98] measured the expression of four calcium signaling lncRNAs, Long intergenic non-protein coding RNA 1231 (LINC01231), Long non-coding RNA solute carrier family 25 member 12 (lnc-SLC25A12), Long non-coding melatonin MT1 receptor (lnc-MTR-1), and Long intergenic non-protein coding RNA 606 (LINC00606), along with their related genes, *Solute Carrier Family 1 Member 1* (*SLC1A1*), *Solute Carrier Family 25 member 12* (*SLC25A12*), *Ryanodine Receptor 2* (*RYR2*), and *ATPase plasma membrane Ca^2+^ transporting 2* (*ATP2B2*), in the blood of ASD and healthy subjects. The authors found differences in the expression of two mRNA-lncRNA pairs: SLC1A1-LINC01231 and RYR2-lnc-MTR-1. The underexpression of *SLC1A1*, which encodes for a glutamate transporter, was accompanied by the upregulation of its cognate lncRNA, LINC01231, in ASD cases compared to controls. The authors also found an overexpression of the calcium ion release channel RYR2 in ASD children, while lnc-MTR-1 tended to be downregulated [98]. Notably, copy number variation in the *RYR2* gene has been reported in ASD [99]. Among the lncRNAs studied, LINC01231 showed the highest diagnostic potential (AUC: 0.75) for distinguishing ASD cases from controls [98] (Table 1).

Mitochondrial dysfunctions and OS can trigger the inflammatory response through the activation of intracellular signaling cascades such as nuclear factor kappa B (NF-κB) and phosphoinositide 3-kinase/AKT serine/threonine kinase (PI3K/AKT), which lead to the expression of numerous pro-inflammatory molecules, including interleukin-6 (IL-6), interleukin-1β (IL-1β), interferon-γ (IFN-γ), and tumor necrosis factor α (TNF-α) [100]. On the other hand, immune cells produce large amounts of ROS, highlighting that OS and inflammation sustain each other in a self-reinforcing vicious cycle [101,102]. In the brain, the activation of microglia—the resident immune cells of central nervous system—can lead to chronic neuroinflammation and alterations in neural signaling and cognitive function [103]. Mounting evidence indicates that inflammation plays a key role in ASD pathogenesis [104,105,106,107,108,109,110,111,112,113]. In light of these observations, several lncRNAs involved in immune–inflammatory pathways have been investigated as possible biomarkers. Xie and collaborators [114], focusing on the innate immune system abnormalities in ASD, observed elevated TNF-α levels in the blood of children with ASD. This prompted them to investigate the potential association of this cytokine with the expression of TNFα and hnRNPL Related immunoregulatory LincRNA (THRIL), an lncRNA involved in the negative regulation of TNF-α via heterogeneous nuclear ribonucleoprotein L (hnRNPL) [115]. Xie and colleagues [114] found that THRIL expression was significantly lower in children with ASD compared to control children. A previous study by Li and colleagues demonstrated that increased THRIL expression in macrophages could regulate TNF-α expression through epigenetic mechanisms, and conversely, that TNF-α could downregulate THRIL expression via a negative feedback loop [115]. Considering these findings, Xie and collaborators [114] proposed that THRIL might play a role in the dysregulation of the TNF-α signaling observed in ASD; however, the diagnostic performance of this lncRNA has not yet been tested.

The interferon γ–antisense RNA (IFNG-AS1) lncRNA, also known as NeST and TMEVPG1 [116,117], regulates the expression of IFN-γ [118], a cytokine involved in immune-related disorders [119]. IFNG-AS1 is located next to the *IFNG* gene on chromosome 12, where it acts as a regulatory enhancer of *IFNG* expression [120]. Fu and colleagues [121], using a BTBR-ASD mouse model, demonstrated that the administration of IFNG-AS1-containing exosomes, derived from mesenchymal stem cells isolated from adipose tissue, improved neurogenesis, suppressed brain inflammatory microenvironment, and ameliorated ASD-like behavior via the miR-21a-3p/PI3K(p110α)/AKT axis (Figure 2). Fallah and collaborators [122] found a decreased expression of IFNG-AS1 in the peripheral blood of ASD children and an inverse correlation between IFN-γ and IFNG-AS1 expression, highlighting an imbalance in their interactive network (Table 1).

Inappropriate activation of NF-κB has been associated with neuroinflammatory responses [123]. Young and collaborators [124] reported that NF-κB is abnormally expressed in the orbitofrontal cortex of ASD subjects, contributing to inflammation and activation of resident immune cells in brain regions associated with behavioral and clinical symptoms of the disease. Honarmand Tamizkar and colleagues [125] postulated that some genes and lncRNAs associated with NF-κB, previously found dysregulated in schizophrenia [126], might also be aberrantly expressed in the peripheral blood of ASD patients. The authors detected an overexpression of three lncRNAs associated with NF-κB, namely Adipogenic differentiation induced non-coding RNA (ADINR), Antisense non-coding RNA in the INK4 Locus (ANRIL), and NF-κB Interacting lncRNA (NKILA), in ASD subjects compared to controls. None of the identified lncRNAs had previously been linked to ASD, except for ANRIL, for which a tendency for some *ANRIL* haplotypes to associate with ASD risk in the Iranian population was demonstrated [127]. The evaluation of the lncRNA suitability in distinguishing between ASD patients and healthy subjects indicated ANRIL as the lncRNA with the best diagnostic power (AUC: 0.857), followed by NKILA (AUC: 0.757) (Table 1).

Taheri’s research group [128] evaluated the expression of Colon Cancer-Associated Transcripts 1 and 2 (CCAT1 and CCAT2) in the whole blood of ASD children and healthy controls, since both lncRNAs are involved in the regulation of immune-related genes and in inflammation [129,130,131,132]. The study revealed significantly reduced expression of CCAT1 and elevated levels of CCAT2 in ASD children compared to controls. ROC analysis showed that CCAT2 had stronger diagnostic performance than CCAT1 (AUC: 0.779 and 0.663, respectively) (Table 1) in distinguishing ASD patients from healthy individuals [128].

**Table 1:** Human dysregulated blood-derived long non-coding RNAs (lncRNAs) in autism spectrum disorders (ASDs).

lncRNAs	Subjects (Gender and Age)	Source	lncRNA Expression	ASD-Related Pathways Suggested by Authors	Molecular Mechanism in ASD	AUC	Sensitivity	Specificity	Ref.
DISC2	30 ASD children (mean age: 6 ± 1.4 years); 41 healthy children (age-, gender-, and ethnicity-matched)	PB	↑	Neuronal differentiation	Unknown	AUC = 0.763 (*p* < 0.0001)	83.33%	73.17%	[53]
PRKAR2A-AS1	30 ASD children (mean age: 6 ± 1.4 years); 41 healthy children (age-, gender-, and ethnicity-matched)	PB	↑	Unknown	Unknown	AUC = 0.794 *p* < 0.0001	86.67%	78.05%	[53]
LOC101928237	30 ASD children (mean age: 6 ± 1.4 years); 41 healthy children (age-, gender-, and ethnicity-matched)	PB	↑	Unknown	Unknown	AUC = 0.9 • (*p* < 0.0001)	90%	82.93%	[53]
LRRC2-AS1	30 ASD children (mean age: 6 ± 1.4 years); 41 healthy children (age-, gender-, and ethnicity-matched)	PB	↓	Unknown	Unknown	AUC = 0.929 • (*p* < 0.0001)	86.67%	100%	[53]
SNHG6	30 ASD children; 41 healthy children (age-, gender-, and ethnicity-matched; age unspecified)	PB	↓	Vitamin D receptor pathway	Interaction with miR-181c	AUC = 0.94 • (*p* = 0.002)	60.00%	73.17%	[57]
lincRNA-ROR	30 ASD children (11 females and 19 males, mean age: 6 ± 1.4 years); 41 healthy children (11 females and 30 males, mean age: 6 ± 1.74)	PB	↓	Neuronal differentiation	Unknown	AUC = 0.85 (*p* < 0.0001)	86.67%	65.85%	[60]
LINC-PINT	30 ASD children (11 females and 19 males, mean age: 6 ± 1.4 years); 41 healthy children (11 females and 30 males, mean age: 6 ± 1.74)	PB	↓	Neuronal differentiation	Unknown	AUC = 0.67 (*p* = 0.0138)	/	/	[60]
lincRNAp21	30 ASD children (11 females and 19 males, mean age: 6 ± 1.4 years); 41 healthy children (11 females and 30 males, mean age: 6 ± 1.74)	PB	↓	Neuronal differentiation	Unknown	AUC = 0.64 (*p* = 0.0394)	/	/	[60]
PCAT-29	30 ASD children (11 females and 19 males, mean age: 6 ± 1.4 years); 41 healthy children (11 females and 30 males, mean age: 6 ± 1.74)	PB	↓	Neuronal differentiation	Unknown	AUC = 0.74 (*p* = 0.0005)	/	/	[60]
PCAT-1	30 ASD children (11 females and 19 males, mean age: 6 ± 1.4 years); 41 healthy children (11 females and 30 males, mean age: 6 ± 1.74)	PB	↓	Neuronal differentiation	Unknown	AUC = 0.84 (*p* < 0.0001)	80%	70.73%	[60]
MEG3	30 ASD children (mean age: 6.01 ± 1.39 years); 41 healthy children (age-, gender-, and ethnicity-matched)	PB	↑	Neuronal synaptic plasticity; apoptotic pathway	Increases CDH2 expression via EP300, repressing neuronal viability *	AUC = 0.792 (*p* < 0.0001)	83.33%	70.73%	[62]
Shank2-AS	40 ASD children; 40 healthy children (age and gender unspecified)	Lymphocytes from PB	↑	Apoptotic pathway	Decreases Shank2 gene expression *	/	/	/	[69]
NEAT1	30 ASD children (mean age: 6.01 ± 1.4 years); 41 healthy children (mean age: 6 ± 1.4 years, gender-matched)	PB	↑	Apoptotic pathway	Interaction with miR-497/BDNF pathway; recruits YY1 to regulate UBE3A expression *	AUC = 0.759 (*p* < 0.0001)	70%	75.61%	[71]
TUG1	30 ASD children (mean age: 6.01 ± 1.4 years); 41 healthy children (mean age: 6 ± 1.4 years, gender-matched)	PB	↑	Apoptotic pathway	Sponge of miR-9	AUC = 0.733 (*p* = 0.0001)	76.67%	65.85%	[71]
	16 ASD children (12 males and 4 females; mean age: 6 ± 1.8 years) 16 healthy controls (11 males and 5 females; mean age: 7.6 ± 2.1 years)	PB mononuclear cells (PBMCs)	↑	Protection against oxidative stress	Regulation of TLDC1 expression	/	/	/	[82]
PVT1	60 ASD children (mean age: 7.07 ± 2.56 years); 58 healthy children (mean age: 7.76 ± 2.67 years)	serum	↓	Neuro- protection	Unknown	AUC = 0.848 (95% CI = 0.78–0.92)	85.0%	79.3%	[90]
LINC01231	30 ASD children (11 females mean age: 6 ± 1.73 years and 19 males, mean age: 6 ± 1.33 years); 41 healthy children (11 females, mean age: 5.63 ± 1.28 years and 30 males, mean age: 6.2 ± 1.88 years)	PB	↑	Ca^2+^ signaling	Unknown	AUC = 0.75 ± 0.06 (*p* = 0.0003)	0.77	0.76	[98]
THRIL	10 ASD children; 41 healthy children (age and gender unspecified)	PB	↓	Immune signaling/ inflammation	Regulation of TNF-α expression	/	/	/	[114]
IFNG-AS1	50 ASD children (15 females and 35 males, mean age: 6 ± 1.4 years); 50 healthy controls (14 females and 36 males, mean age: 6 ± 1.74 years)	PB	↓	Immune signaling/ inflammation neurogenesis	Regulation of IFNG; regulation of miR-21a-3p/PI3K (p110α)/ AKT axis *	/	/	/	[122]
ADINR	30 ASD children (11 females and 19 males, mean age: 6 ± 1.39 years); 41 healthy children (11 females and 30 males, age- and ethnicity- matched)	PB	↑	Immune signaling/ inflammation: NF-κB pathway	Unknown	AUC = 0.735	/	/	[125]
ANRIL	30 ASD children (11 females and 19 males, mean age: 6 ± 1.39 years); 41 healthy children (11 females and 30 males, age- and ethnicity- matched)	PB	↑	Immune signaling/ inflammation: NF-κB pathway	Unknown	AUC = 0.857	/	/	[125]
NKILA	30 ASD children (11 females and 19 males, mean age: 6 ± 1.39 years); 41 healthy children (11 females and 30 males, age- and ethnicity- matched)	PB	↑	Immune signaling/ inflammation: NF-κB pathway	Unknown	AUC = 0.757	/	/	[125]
CCAT1	30 ASD children (11 females and 19 males mean age: 6.01 ± 1.39 years); 41 healthy controls (11 females and 30 males (age-, sex-, and ethnicity-matched)	PB	↓	Immune signaling/ inflammation	Unknown	AUC = 0.663 (*p* = 0.016)	54.33%	82.93%	[128]
CCAT2	30 ASD children (11 females and 19 males mean age: 6.01 ± 1.39 years); 41 healthy controls (11 females and 30 males (age-, sex-, and ethnicity-matched)	PB	↑	Immune signaling/ inflammation	Unknown	AUC = 0.779 (*p* < 0.0001)	86.67%	73.17%	[128]

“↑” means upregulated lncRNAs in ASD; “↓” means downregulated lncRNAs in ASD; PB, peripheral blood; CDH2, cadherin2; EP300, E1A binding protein p300 transcription factor; BDNF, brain-derived neurotrophic factor; YY1, Yin-Yang 1 transcription factor; UBE3A, ubiquitin protein ligase E3A; TLDC1, TBC/LysM-associated domain containing 1; TNF-α, tumor necrosis factor α; IFNG, interferon gamma; PI3K/AKT, phosphoinositide 3-kinase/AKT serine/threonine kinase; NF-κB, nuclear factor kappa B. Ref. means references; * marks the only validated markers in ASD; • marks highly satisfactory discriminatory accuracy.

### 2.2. LncRNAs Selected by High-Throughput Analysis

The first attempt to identify a genome-wide differential expression of lncRNAs in ASD blood specimens was carried out by Wang and colleagues [70]. In a Chinese cohort, they compared RNA extracted from peripheral leukocytes of ASD and control children and identified 3929 differentially expressed lncRNAs—2407 upregulated and 1522 downregulated. Functional pathways analysis revealed that deregulated lncRNAs were mainly involved in infection and inflammation pathways, while the upregulated ones were implicated in neurological regulatory pathways, such as long-term depression, long-term potentiation, and synaptic vesicle cycling. In particular, Wang and colleagues [70], reported several dysregulated synapsis-associated lncRNAs in ASD peripheral leucocytes, some of which reside within genes that code for proteins involved in synaptic vesicle transportation and cycling. Notably, these lncRNAs were also analyzed by Fang and colleagues in circulating exosomes isolated from peripheral blood, and they found some to be differentially expressed in ASD subjects compared to controls [133]. In addition, Wang and coworkers [70] identified many dysregulated lncRNAs located at loci where rare high-impact genetic variants are known to increase ASD risk. Since many of these lncRNA originate from antisense, bidirectional, and intragenic traits of homeobox (HOX) genes, the authors proposed that HOX-related lncRNAs could represent a novel group of potential ASD biomarkers. The differential expression of two lncRNAs, named Shank2-AS and BDNF antisense RNA (BDNF-AS), which are the antisense transcripts of the *SHANK2* and *BDNF* genes, respectively, confirms that exploring lncRNAs at ASD-related loci may lead to the identification of new ASD potential markers [70].

Recently, exploiting the huge number of omics datasets, other approaches were employed to find putative biomarkers. Sabaie and colleagues [134] used a bioinformatic approach on microarray dataset to discover lncRNAs-associated ceRNA networks in the peripheral blood of ASD subjects. According to the ceRNA hypothesis, a pool of both coding and non-coding RNAs—including lncRNAs, mRNAs, and circular RNAs– compete for miRNA binding through their miRNA response elements (MREs) [30,58,135], establishing interactive networks [136]. Sabaie and colleagues [134] identified three lncRNAs, named Long intergenic non-protein coding RNA 472 (LINC00472), ANP32A intronic transcript 1 (ANP32A-IT1), and RBM26 antisense RNA 1 (RBM26-AS1), within four different ceRNA networks related to immune response in ASD pathogenesis: LINC00472/hsa-miR-221-3p/PTPN11, ANP32A-IT1/hsa-miR-182-5p/S100A2, LINC00472/hsa-miR-132-3p/S100A2, and RBM26-AS1/hsa-miR-182-5p/S100A2. The three lncRNAs identified were associated with ASD for the first time.

Balasubramanian and colleagues, using brain transcriptomic (RNA-Seq) datasets, predicted the miRNA sponge modules associated with pan-neuropsychiatric disorders, including ASD. Some of these modules were also confirmed in blood transcriptomic dataset from ASD subjects, leading to the identification of two lncRNAs, RP11-448G15.3 and WAC-AS1, as potential ASD biomarkers (AUC > 0.70) [137].

The ceRNA network approach was also used by Jiang and collaborators [138]; by integrating multiple omics data from ASD postmortem brain tissue samples, the authors built a ceRNA network where the MIR600 host gene (MIR600HG) lncRNA emerged as a key player. This lncRNA was consistently downregulated not only in brain tissues but also in peripheral blood, suggesting its putative role as an ASD biomarker. Based on its downstream mRNA targets, the authors suggested that MIR600HG may impair synaptogenesis in ASD, offering novel insights into the molecular mechanisms of the disorder.

## 3. Discussion

**Figure 3:**
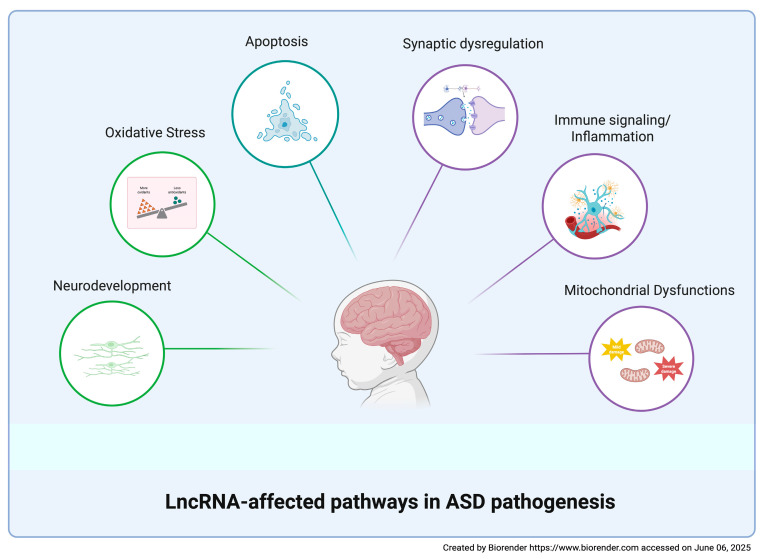
LncRNA-affected pathways in ASD pathogenesis. A schematic representation of the main biological pathways affected by dysregulated lncRNAs in ASD.

The diagnosis of ASD is currently based only on clinical observation; it usually occurs around three years of age and it may be further delayed due to the large heterogeneity of ASD symptoms [12]. It should be emphasized that early diagnosis can anticipate potential therapeutic interventions during a time window when neuronal plasticity allows for the modification of adverse developmental trajectories [19]. The identification of reliable ASD markers would not only facilitate early diagnosis but also contribute to the discovery of possible therapeutic targets highly demanded in a disorder for which no effective interventions have been found so far. The extensive clinical and biological heterogeneity complicates the search for biomarkers. Although several biomolecules have been proposed, to date, no ASD biomarkers meet the standards required to inform clinical trials [139]. In this review, we focused on dysregulated human blood-derived lncRNAs as potential ASD biomarkers. Owing to their specificity and stability, lncRNAs represent an attractive diagnostic tool for clinical use, especially when isolated from peripheral blood, an accessible tissue requiring cost-effective experimental handling. The identified dysregulated blood-derived lncRNAs are mainly involved in neurodevelopment, synaptic dysregulation, apoptosis, mitochondrial dysfunctions, OS, immune signaling, and inflammation, thus indicating these pathways as candidates for diagnostic interventions in ASD (Figure 3 and Table 1). Although lncRNAs have been suggested to be involved in ASD-related pathways by the authors, limited information has been gathered about the involved molecular mechanisms. Functional studies in cellular or animal ASD models have been carried out only for MEG3, NEAT1, Shank2-AS, and IFNG-AS1 (Figure 2).

In most of the studies reviewed here, transcripts were isolated from total blood, which contains either lncRNAs derived from blood cells or released by other tissues. Some of these lncRNAs are packaged and protected within extracellular vesicles (EVs), nano-sized lipid bilayer particles that reflect the features of the originating tissue. The possibility to isolate brain-derived EVs, which contain molecules related to neuronal functions, paves the way for the detection of reliable markers for neurological diseases by opening a non-invasive molecular window into the brain. Using this strategy, Qin and colleagues [140] identified more than 1700 dysregulated lncRNAs in neuronal-cell-derived EVs in ASD which could be evaluated as candidate biomarkers for diagnostic purposes.

Although aberrant expression is a prerequisite for the selection of putative biomarkers, both specificity and sensitivity are critical requirements for their translation to clinical practice. Many of the studies listed here included ROC analyses for the identification of lncRNAs with sufficient sensitivity and specificity to support ASD diagnosis [141,142]. Notably, the diagnostic power of ROC analysis should not be immediately translated into the clinical context since it only assesses the ability of the tested parameter to discriminate between the two examined populations. Although several lncRNAs showed acceptable AUC values, a satisfactory discriminatory accuracy was achieved only for LOC101928237, LRRC2-AS1, and SNHG6 [53,57]. Diagnostic power could be improved by combining multiple markers to identify a diagnostic signature [143]. This integrated approach has been rarely explored for lncRNAs in ASD research. For instance, a superior diagnostic performance was achieved by combining the expression levels of PVT1 and its target miR-21-5p, with respect to the diagnostic power of each single molecule [90]. Recently, the integration of multiple extensive omics studies has led to the establishment of networks based on lncRNA, mRNA, and miRNA interactions (ceRNAs), which could be employed in clinical practice following a proper validation. Currently, only three studies have investigated the role of ceRNA networks in ASD blood samples [134,137,138]; therefore, this field deserves to be explored.

It is essential to recognize that candidate biomarkers are expected to provide reliable and systematic evidence demonstrating the clinical relevance of the selected lncRNAs for disease diagnosis. Considering this, several important limitations emerge from the reviewed studies. Most of them should be considered as discovery studies, as no subsequent validation was performed to confirm the preliminary results. Every putative biomarker should undergo validation that demonstrates the robustness of results in separate cohorts to prevent sporadic findings and avoid the introduction of tests based on presumed worthless biomarkers. Only a limited number of lncRNAs have been evaluated in independent populations. In particular, the upregulation of Shank2-AS observed in ASD leukocytes was confirmed in peripheral blood lymphocytes of another cohort of ASD subjects [69,70]. In addition, TUG1 was found to be upregulated in both PBMCs and in peripheral blood of ASD subjects from two independent populations [71,82]. Furthermore, the expression of DISC2 was investigated in two independent ASD groups, yielding inconsistent results across studies [53,54]. It should be noted that there is very little information about the demographic and diagnostic criteria, such as symptom severity, co-morbidities, inheritance, and environmental conditions. All these issues are sources of variability, which may account for the lack of reproducibility of the findings. Moreover, sample preparation and RNA processing methods differ across the examined studies. In particular, since lncRNAs are relatively low in abundance in blood samples, bulk detection methods, such as microarrays and NGS, should be preferentially confirmed using more sensitive techniques, like RT-PCR.

Most of the examined studies are based on small sample sizes and single-center cohorts, which inevitably limit the statistical power. Notably, eighteen out of the twenty-three differentially expressed lncRNAs reported in Table 1 were studied by the same research group and were apparently evaluated in the same cohorts of subjects. A few inaccuracies (e.g., inconsistency between AUC values reported in the text with those reported in the figures) make it difficult to assess the relevance of the reported data. The geographical specificity of the recruited subjects further restricts the applicability of these results to broader patient populations with diverse ethnic backgrounds. 

In the studies reviewed here, no data are available on the ability of lncRNAs to distinguish ASD subjects from patients affected by diseases which share some of the ASD features. In fact, several dysregulated lncRNAs that emerged as ASD putative biomarkers have also been observed in the peripheral blood of subjects affected by other neurological disorders; for instance, TUG1 has been proposed as a biomarker for the diagnosis of temporal lobe epilepsy [144], while PVT1 is downregulated in schizophrenia [88], and THRIL is underexpressed in epilepsy [145]. Therefore, it would be necessary to estimate the performance of the proposed lncRNA in distinguishing ASD subjects from patients with other neurological disorders to clarify their effective application in clinical practice. 

Although these limitations compromise the reliability and the generalization of the conclusions, some of the lncRNAs summarized here are worthy of interest for ASD biomarker development. For instance, MEG3, NEAT1, Shank2-AS, and IFGN-AS1 are relevant due to their experimentally observed implication in ASD pathogenesis, while both TUG1 and Shank2-AS are also important for their expression patterns, which have been consistently reported in more than one study, although a more effective confirmation would be needed. Moreover, LOC101928237, LRRC2-AS1, and SNHG6 seem to be promising according to their AUC values. However, data concerning all lcnRNAs reported here should be considered as preliminary findings deserving deeper exploration to assess their effective translational potential in order to move the field forward.

## 4. Conclusions and Future Directions

This review highlights the important role of blood-derived lncRNAs in ASD, although this field is still in the early stages of development. Independent studies should be carried out in larger cohorts of subjects with gold-standard enrollment criteria in order to corroborate the reproducibility of the current results. In addition, since many potential biomarkers are altered in several diseases, the obtained results should be validated to test their specificity in subjects affected by similar neurodevelopmental and systemic disorders. Moreover, it would be useful to extend the analysis to younger cohorts to identify whether relevant lncRNAs are expressed in very young children so as to be used as biomarkers for early diagnosis. Finally, the validation of specific biomarkers might also benefit from longitudinal cohort studies where ASD subjects are followed over time across their lifespan. A significant contribution to biomarker discovery could derive from omics approaches, including transcriptomics, proteomics, epigenetics, and metabolomics. The integration of multiple omics would make it possible to set a network of molecular interactions disclosing potential molecular mechanisms which are altered in ASD. It is known that most biomarkers currently applied in clinical practice stem from the deep knowledge of their molecular mechanisms in the pathology, which highlights the need to encourage basic research. To this purpose, new approaches employing cellular and animal ASD models, along with CRISPR-based strategies to selectively modulate the expression of a candidate lncRNA, could improve the assessment of its roles in ASD-related pathways. The CRISPR/Cas system provides innovative tools to investigate the potential roles of lncRNAs through knockout, knockdown, overexpression, and imaging approaches (for a comprehensive overview, see [146,147,148]). This technology has been successfully applied to study the function several lncRNAs. For example, by using a combination of cellular reprogramming and genome editing, it has been shown that the disruption of PTCHD1-AS impairs excitatory neurotransmission in ASD [149]. By using the CRISPR/Cas system, it is possible to modulate the expression of specific lncRNAs in order to analyze the changes in transcriptome, proteome, and metabolome, thus highlighting the downstream effects of lncRNA regulation [150]. For instance, the deletion of GOMAFU promoter through the CRISPR-Cas9 tool allowed for identifying transcriptomic alterations in numerous genes involved in IFN signaling [151]. Moreover, the integration of lncRNA expression profiles with complementary datasets, including quantitative proteomics and high-resolution imaging, could help decipher cellular functions and the spatial and temporal regulation of lncRNAs [152].

In conclusion, our review suggests that blood-derived lncRNAs deserve further studies aimed at exploiting their potential as candidate ASD biomarkers.

## Data Availability

Data sharing is not applicable.
